# Variation in benthic long-term data of transitional waters: Is interpretation more than speculation?

**DOI:** 10.1371/journal.pone.0175746

**Published:** 2017-04-19

**Authors:** Michael Lothar Zettler, René Friedland, Mayya Gogina, Alexander Darr

**Affiliations:** Leibniz Institute for Baltic Sea Research Warnemünde, Seestr. 15, Rostock, Germany; Technical University of Denmark, DENMARK

## Abstract

Biological long-term data series in marine habitats are often used to identify anthropogenic impacts on the environment or climate induced regime shifts. However, particularly in transitional waters, environmental properties like water mass dynamics, salinity variability and the occurrence of oxygen minima not necessarily caused by either human activities or climate change can attenuate or mask apparent signals. At first glance it very often seems impossible to interpret the strong fluctuations of e.g. abundances or species richness, since abiotic variables like salinity and oxygen content vary simultaneously as well as in apparently erratic ways. The long-term development of major macrozoobenthic parameters (abundance, biomass, species numbers) and derivative macrozoobenthic indices (Shannon diversity, Margalef, Pilou’s evenness and Hurlbert) has been successfully interpreted and related to the long-term fluctuations of salinity and oxygen, incorporation of the North Atlantic Oscillation index (NAO index), relying on the statistical analysis of modelled and measured data during 35 years of observation at three stations in the south-western Baltic Sea. Our results suggest that even at a restricted spatial scale the benthic system does not appear to be tightly controlled by any single environmental driver and highlight the complexity of spatially varying temporal response.

## Introduction

The usage of long-term data to analyse anthropogenic, climatic or cosmic influence on our environment is very common within all associated fields of research (e.g., [1]). In the last few decades, even centuries [2], long-term monitoring programs have been launched in more or less all inhabited (atmospheric, terrestrial and aquatic) and not inhabited spaces (deep subsurface, outer space) to measure physical, chemical and biological variables. It always poses a challenge to analyse the gathered data in an appropriate way to ensure that the gathered information is directly or indirectly linked to effects or influences on our environment. Further, the potential impact of these changes or modifications on human life with all its facets (health, nutrition, wellbeing) should be detectable promptly or, ideally–in advance.

Four decades ago the HELCOM (Baltic Marine Environment Protection Commission, Helsinki Commission) was established to protect the marine environment of the Baltic Sea from all sources of anthropogenic pressures through intergovernmental cooperation. Regionally coordinated monitoring of physical, chemical and biological variables was established in 1979 as a key component in the HELCOM work plan. These monitoring programs are the source of data for the analysis of (natural) long-term trends and thereby the backbone for many indicator-based assessments of the state of and pressures on the marine environment [3].

Germany as one of the HELCOM’s contracting parties started its coordinated monitoring activities in 1980. From a macrozoobenthic perspective, after 35 years of measuring and observation the gained data pool can be regarded as a treasure. The central part is the yearly autumnal record of endobenthic macrofauna with its three components: taxonomic diversity, abundance and biomass.

The wide use of benthic macroinvertebrate communities to assess water quality is promoted by the fact that pollution tolerance of many taxa is well-documented allowing biological indices to be developed [4]. Further, the sampled macroinvertebrate community integrates the state of the environment over the previous months to years [5]. But first of all, variations in communities are a result of the interaction of the various ecosystem components, both living and non-living. Ideally, the natural spatial and temporal variability of macroinvertebrate communities should be analysed and comprehended without the disturbance of human activities. However, since nowadays nearly all marine systems, and especially the coastal areas of densely populated regions such as the southern Baltic, suffer at least some degree of anthropogenic pressures, the interpretation of long-term data may be the only approach to provide the requisite insights.

The German part of the Baltic Sea is located in an area with highly variable abiotic conditions, where frequently saline North Sea water is flowing in and brackish water from the central Baltic is flowing out. Salinity and oxygen supply, regarded as the main environmental parameters for the inhabitants in these transitional waters [6, 7], vary on seasonal, annual and perennial scales. Since many benthic species in the Baltic Sea live at the limit of their physiological tolerance [8–10], even minor and *a fortiori* major environmental changes are likely to induce large community changes.

The driving forces behind these key parameters for marine coastal ecosystems include eutrophication and climate change [11]. Both interact with the abiotic parameters in several ways and are directly or indirectly responsible for the changing environment [12–15]. The North Atlantic Oscillation, whose index (NAOI) integrates several climatic variables (e.g., water temperature, prevailing wind direction and speed, precipitation) [14] is often used to detect these changes. Variations in biomass, abundance, community structure, and functioning of benthic systems directly or indirectly related to variability in the winter NAO index have been analysed in different areas over recent decades (e.g., [15–23]).

The biggest challenge in a highly variable area in a potentially multi-force driven system is to find any significant relationships between abiotic and biotic parameters and to identify predictors relevant for changing zoobenthic communities on different temporal scales. Facing and overcoming this challenge is essential for the assessment of the impact of anthropogenic activities as required by European legislation, e.g. in the Marine Strategy Framework Directive (MSFD).

Therefore, we address several questions in our study: (1) Can the temporal variability of macrozoobenthic data be explained using statistical approaches? Are the results reasonable at all? (2) Are the high fluctuations of diversity, abundance and biomass of macrozoobenthos in transitional waters like the southern Baltic Sea mainly driven by coincidence or by principles? (3) Can any temporal trends, regime shifts or changes be explained by abiotic variables or climate change?

## Material and methods

### Investigation area

**Fig 1:**
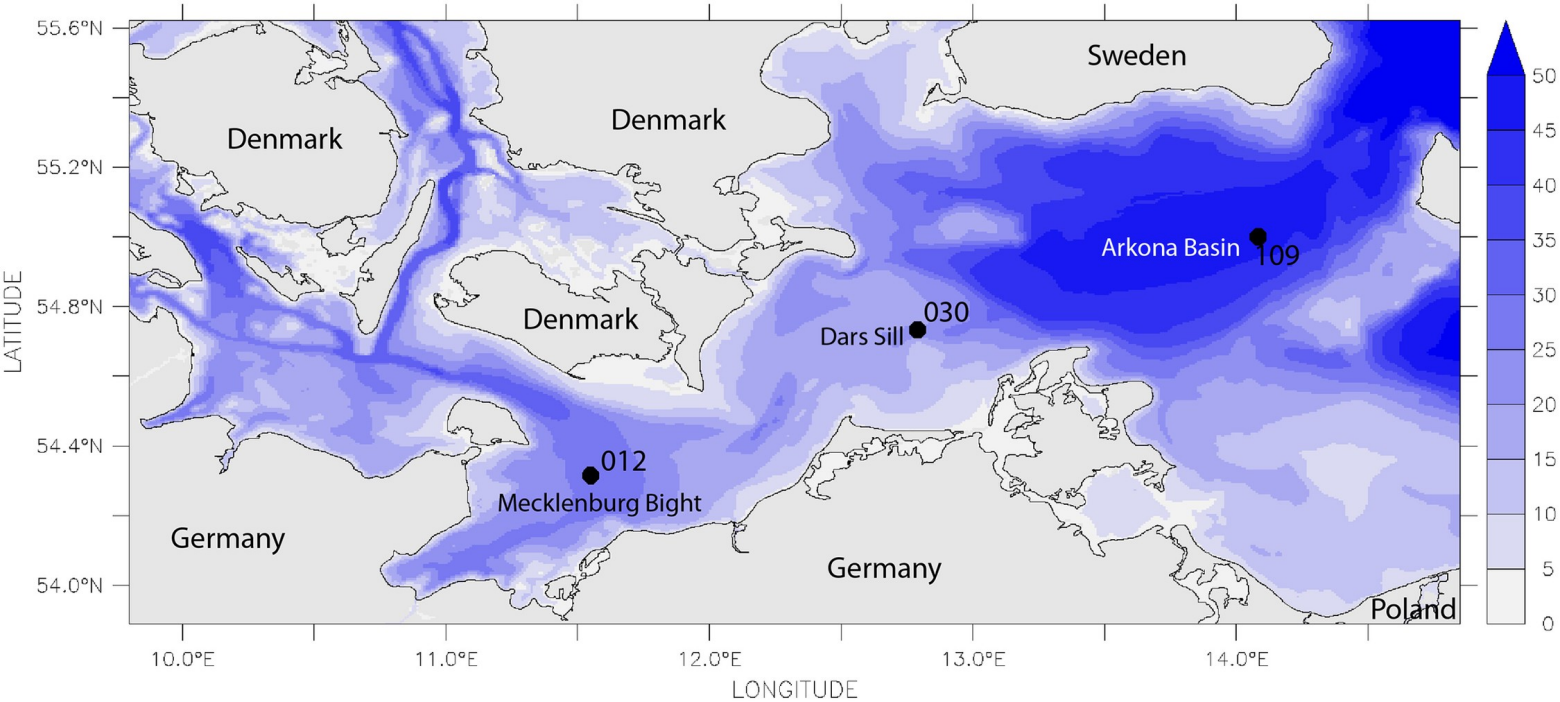
Bathymetry (water depth in m) of the southern Baltic Sea with three selected long-term monitoring stations.

**Fig 2:**
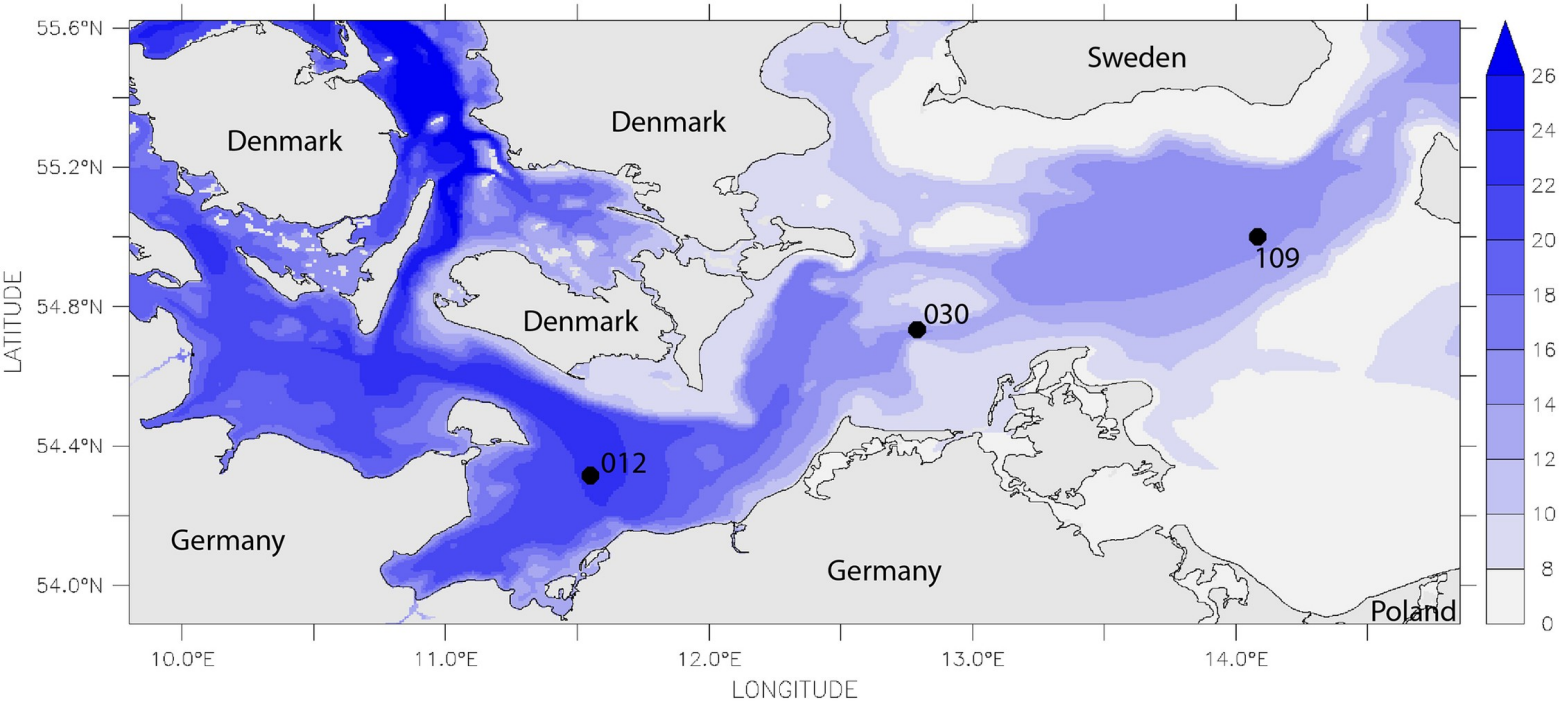
Median of bottom salinity calculated from model simulations covering the last 15 years.

The Baltic Sea, formed after the latest glaciation, is a young ecosystem continuously undergoing postglacial successional changes [24]. It is an enclosed, nontidal ecosystem with steep latitudal and vertical salinity gradients maintained by the advection of saline North Sea water via barotropic and baroclinic inflows [25]. The south-western parts including the Belt Sea are closely connected to the Kattegat and Skagerrak and show salinities between 25 and 30 psu (Figs 1 and 2). Within a few 100 km eastwards the salinity drops down to 8 psu. Consequently, the number of marine species declines significantly [9, 10, 26]. Oxygen availability also limits species distribution, as most benthic organisms are sensitive to long-term low oxygen conditions [27, 28]. Therefore, in the deeper basins below the halocline, benthic life is often absent (Gotland Basin) or impoverished (e.g., Arkona Basin), particularly after longer periods without saline water inflows. In the shallow parts of the Baltic (e.g., Mecklenburg Bight) hypoxia may occur during the summer months due to a strong stratification.

**Fig 3:**
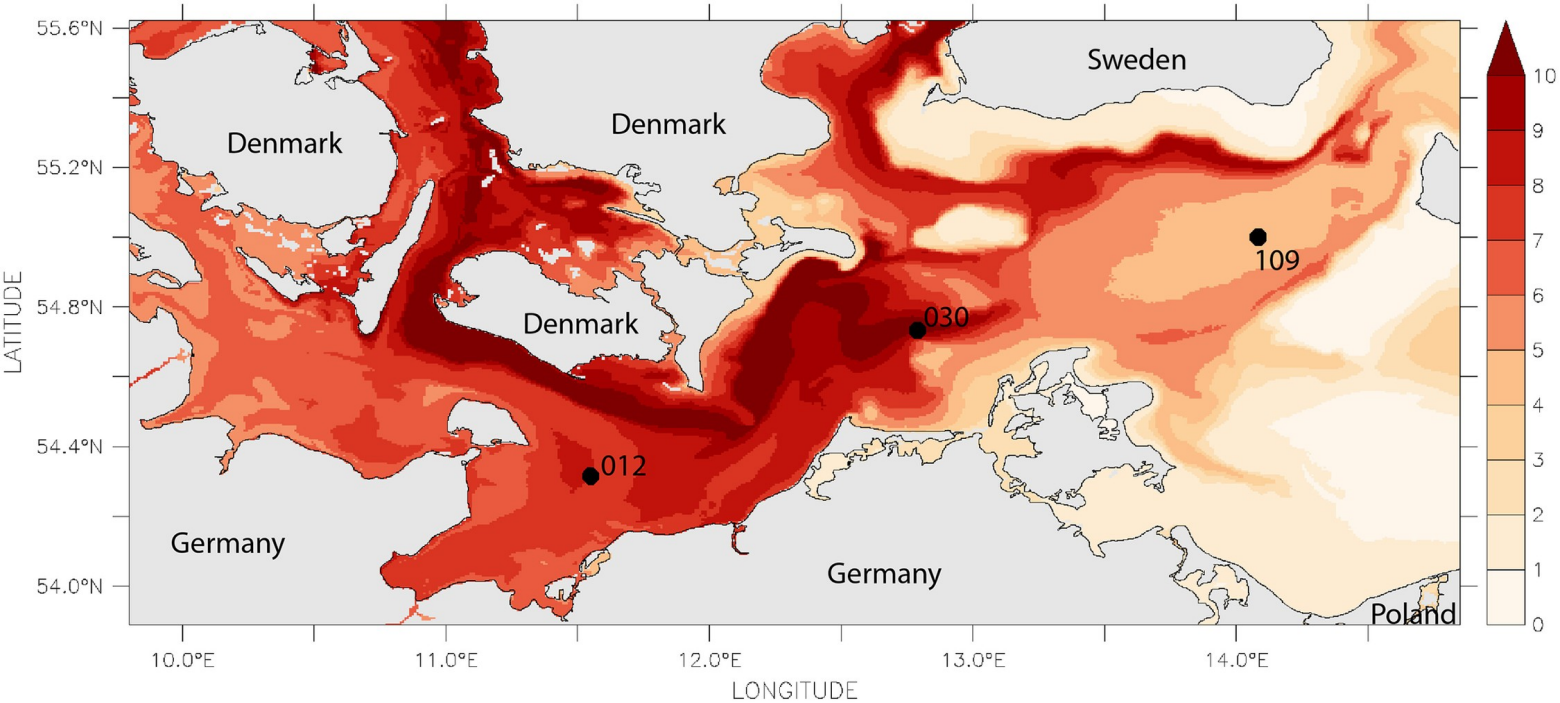
Difference of 10th-and 90th-percentile of simulated bottom salinity calculated over the last 35 years.

**Fig 4:**
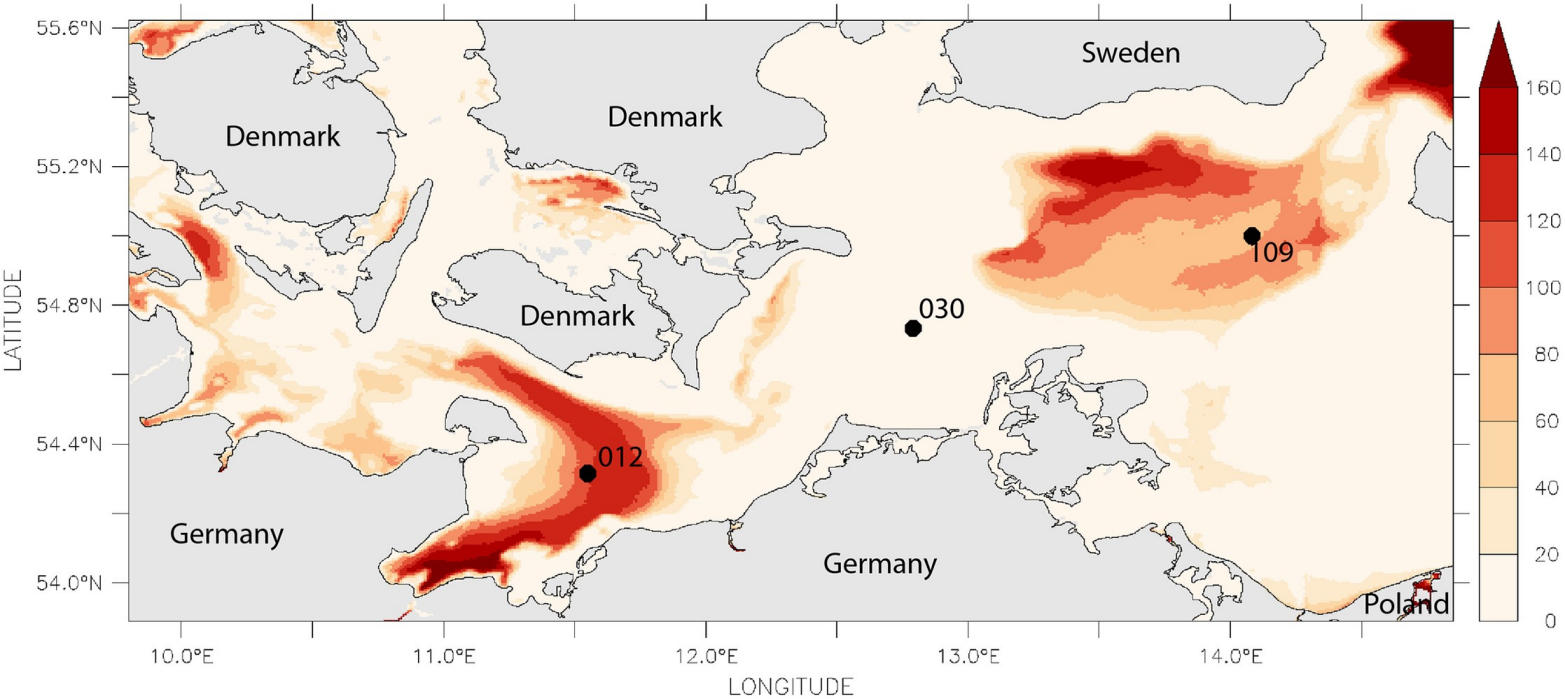
Estimated number of days per year with oxygen concentration below 1 ml/l covering the last 35 years.

Three stations were selected for the present study in order to represent a wide range in salinity, oxygen supply and sediment characteristics (Figs 1–4, Table 1). Station 012 is located in the central Mecklenburg Bight with water depths around 24 m and organic enriched muddy sediments. The easternmost station 109 in the Arkona Basin has similar sediments, but differs in water depth (48 m). The shallowest station 030 (depth 22 m) is located at the Darss Sill and is characterised by well sorted, organic poor, fine to medium-fine sand (median grain size 220 μm). This station is located at the main route of salt-water-inflows. Despite the lowest mean salinity (11.2 psu, Table 1) it shows the strongest salinity variations (Fig 3). The mean duration of oxygen depletion is highest in the Mecklenburg Bight (station 012) and the Arkona Basin (station 109) (Fig 4). The Mecklenburg Bight shows low oxygen conditions every autumn when stratification is strongest, and the Arkona Basin mainly depends on the inflow of oxygen-rich waters.

**Table 1:** Measured abiotic characteristics of the three selected monitoring stations: sediment parameters (organic content, median grain size, fraction smaller 63 μm), chemistry of near bottom waters (mean oxygen content, salinity) and water depth (all given with their standard deviations = SD), only autumn measurements between 2006 and 2015 are used.

	Mecklenburg Bight (012)		Darss Sill (030)		Arkona Basin (109)	
	median	SD	median	SD	median	SD
organic content (%)	9.3	0.6	0.3	0.1	11.9	1.9
median grain size (μm)	18	7	222	4	18	5
<63 μm (%)	94	29	1	2	97	30
oxygen (ml/l)	5.41	0.99	6.60	0.51	3.84	0.97
salinity	19.4	2.1	11.2	3.6	16.3	2.8
depth (m)	24.5		22.5		48	

### Benthos monitoring data

The three selected stations (Fig 1, Table 1) were sampled over the past 35 years as part of the regular HELCOM monitoring program. No specific permissions were required for these locations and activities and no endangered or protected species were involved. At each station, three replicates were taken with a 0.1 m^2^ van Veen grab annually in autumn. All samples were sieved through a 1 mm screen and animals were preserved in the field with 4% formaldehyde. For sorting in the laboratory, a stereomicroscope with 10–40 x magnification was used. All macrofauna samples were identified to the lowest taxonomic level possible. The nomenclature was checked following the World Register of Marine Species (www.marinespecies.org). Some taxonomic groups (e.g., turbellarians, nemerteans, oligochaetes) were lumped due to the changing taxonomic expertise over time and therefore their diversity is likely to be underestimated.

### Oceanographic monitoring data

Derived from the monitoring and IOW`s database [29], observations of basic abiotic parameters were utilized to compute annual parameters, later used as independent variables (e.g. annual minimum and maximum of salinity, minimum oxygen concentration). Oxygen and salinity were measured near the bottom using a profiling CTD-system five to ten times per year during all seasons. Oxygen sensors were calibrated by immediate potentiometric Winkler titration in the ships laboratory. Additionally, Winter-NAO was used as an independent variable, fluctuating on perennial scales but seen as one of the driving forces of the regime shift end of 1980s [30]. We followed the calculation suggested by Climate Research Unit (https://crudata.uea.ac.uk/cru/data/nao).

### Modelling

**Fig 5:**
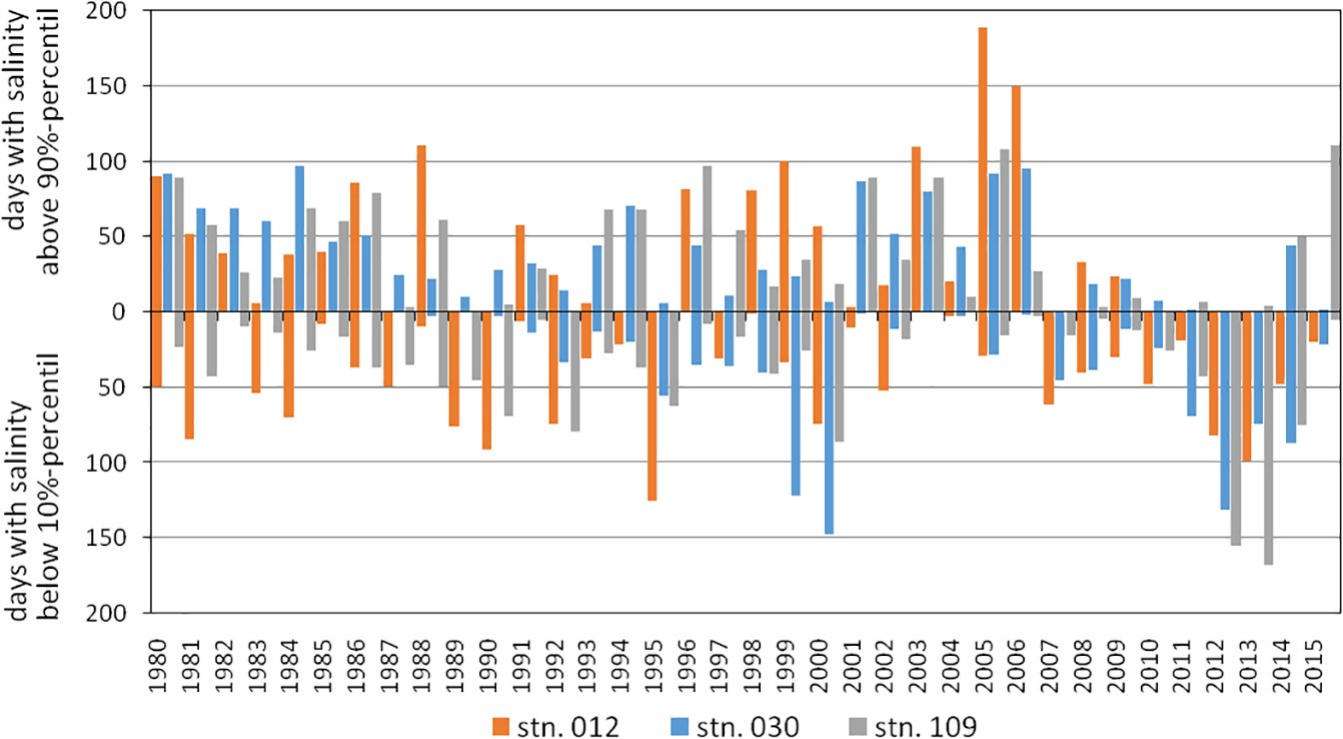
Estimated number of days per year and station with below 10th- and above 90th-percentile of bottom salinity. Simulations covering the last 35 years [stn. 012: p10th (18.41 psu), p90th (25.94 psu); stn. 030: p10th (9.45 psu), p90th (19.29 psu); stn. 109: p10th (12.73 psu), p90th (17.74 psu)].

Observational data on oxygen and salinity were complemented by modelled data to incorporate their variability in the analyses. Both were derived from 3-d simulations using the biogeochemical model system ERGOM, coupled with a hydrographical model. Validation data and details on the model systems are shown by [31–34]. To estimate the salinity variability, the 10th- and 90th-percentiles were computed over the entire simulation period and the number of days per year above the 90th-percentile and below the 10th-percentile were calculated. The horizontal gradient as well as the difference in variability is visible for the whole area (Figs 2, 3 and 5). Using the modelled salinity data, the monthly mean and its deviation from the median (computed for all years) were calculated. In a second step, these monthly salinity anomalies were condensed by computing the annual mean and maximum of the anomalies.

**Fig 6:**
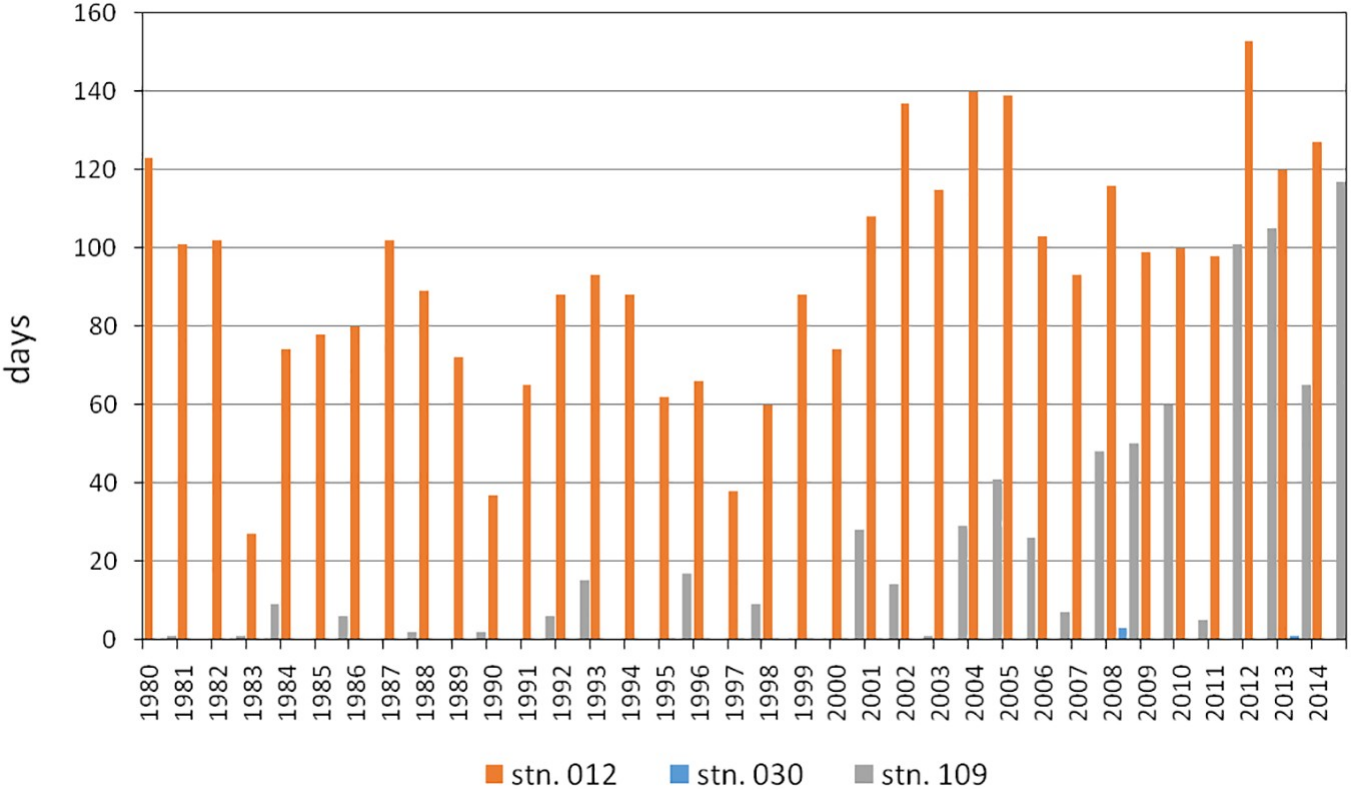
Estimated number of days per year and station with oxygen concentration below 1 ml/l. Simulations covering the last 35 years.

Hypoxia thresholds vary greatly across marine benthic organisms, which cannot be adequately captured by a single, universal threshold [35]. The threshold of hypoxia proposed in the literature range broadly from 0.2 ml/l to 2.8 ml/l; most reports refer to a value of 1.4 ml/l (or 2 mg/l; see [35] and references therein). We included both the measured values and days per year with modelled oxygen concentration below 1 ml/l and 2 ml/l as well as oxygen saturation below 20% (and 50%) as proxies for oxygen-depletion stress in our analysis. To illustrate the station-specific intensity, the days with oxygen concentrations below 1 ml/l are shown in Fig 6.

### Data processing and statistical analysis

The identified, counted and weighed species per replicate were used to calculate the abundance, biomass and species richness per square meter. Besides these basic zoobenthic parameters, we calculated several derivative indices: (1) Shannon diversity index (H'log_2_) [36, 37], (2) Margalef‘s index (*d*) [38], (3) Pilou’s evenness index (J') [39], and (4) Hurlbert index (ES_50_) [40]. Details and equations for each index are given in S1 File. For all basic macrozoobenthic parameters and their derivates (indices) the long-term median value was estimated. The log_10_-transformed ratio *R* between the annual and the long-term median (“anomaly”) was calculated to emphasise the temporal changes or trends:

$$R=\log_{10}(A/M),$$

where *A* is the annual value and *M* is the long-term median value.

To determine the set of variables that best explain the variation of each macrozoobenthos parameter at each station separately, we performed a distance-based linear model permutation test (DistLM) [41] employing the routine from the software PRIMER 6 with PERMANOVA+ add-on. Taking into account that macrofauna can be affected by the previous winter’s conditions [16, 30, 42], we included not only the environmental drivers corresponding to the year of macrofauna sample collection in the test, but also the previous year’s values, treated as additional independent variables. The summarising list of all abiotic variables tested and short accompanying descriptions are provided in S1 Table.

Every variable is treated separately and independently at each station. Statistics are calculated separately for each basic (density, biomass, species richness) and derived (Shannon, Margalef, Pilou, ES_50_) zoobenthic index with the set of variables for the particular station. DistLM analyses were performed after removing highly correlated independent environmental variables (Draftsmans plot, lrl ≥ 0.90). The variables excluded were for station 012 oxygen-days below 20%; for station 030 oxygen-days below 2 ml/l and for station 109 oxygen-days below 2 ml/l, oxygen-days below 50%, oxygen-days below 20%.

Up to 22 independent variables were included in the statistical models (S1 Table). Some variables like “days with salinity above 90th-percentile”, “oxygen-days below 50%”, “days with salinity below 10th-percentile” were square root transformed to remove right-skewness in the raw data in case it was observed on Draftsmans plots. Predictor variables were subjected to a sequential step-wise selection procedure using the Akaike’s information criterion with a correction for finite sample size (AICc [43]). To calculate resemblance in DistLM, Euclidean distance was used for univariate datasets [44]. log_e_ (x+1)-transformation was performed for abundance and biomass response variables. The variables indicating significance levels above the threshold (p>0.05) were excluded from the final model.

Changes in the species composition of the assemblage between samples (years) were assessed by the calculation of Bray-Curtis similarity on log_10_ (x+1)-transformed abundance and subsequent ordination using non-metric multidimensional scaling (nMDS).

## Results

The overall community structure, basic macrozoobenthic parameters and their derivates (indices) differ considerably between the stations (Table 2). Although some species were representative for two or all three stations (e.g. *Diastylis rathkei*), some highly frequent and abundant species were station-specific (Table 3). In the Mecklenburg Bight (012) the ocean quahog (*Arctica islandica*) and the polychaetes *Lagis koreni* and *Nephtys hombergii* were most frequent and dominant. The community at Darss Sill (030) was dominated by the bivalves *Astarte borealis* and *Mytilus edulis* and the gastropod *Peringia ulvae*. In contrast, the Arkona Basin (109) was mainly colonised by the bivalve *Macoma balthica* and the polychaete *Scoloplos armiger*. Long-term abundance was lowest in the Arkona Basin and both highest as well as most variable at the Darss Sill, where the highest median and maximum values of biomass and species richness were also observed. Accordingly, at the Darss Sill station the diversity indices (Margalef, Hurlbert and Shannon) were highest, whereas the evenness index (Pilou) was lowest, indicating a more uneven distribution pattern of species abundance.

**Table 2:** Some biotic characteristics of the three selected monitoring stations. Median values of abundance, biomass, species number and some biotic indices are listed with minimum and maximum measured autumnally over the last 35 years.

	Mecklenburg Bight (012)		Darss Sill (030)		Arkona Basin (109)	
	median	min/max	median	min/max	median	min/max
abundance (ind./m^2^)	499	82/3887	3333	74/12.719	314	18/1075
biomass (g afdw/m^2^)	12.8	2.89/51.52	15.3	1.07/67.13	1.1	0.2/8.2
species number	15	4/29	27	5/38	9	3/17
Margalef (d)	2.29	0.69/3.62	2.93	0.93/4.61	1.57	0.60/2.75
Pilou (J')	0.68	0.42/0.88	0.64	0.30/0.85	0.73	0.21/0.93
Hurlbert (ES_(50)_)	9.52	3.91/11.88	10.33	4.54/15.19	7.39	3.00/12.57
Shannon (H'log2)	2.76	0.99/3.25	2.94	1.12/3.77	2.29	0.49/3.42

**Table 3:** Relevant species with frequency >70% and high abundance or biomass. The most important species per station are listed (B = bivalve, C = cumacean, G = gastropod, P = polychaete).

Mecklenburg Bight (012)		Darss Sill (030)	Arkona Basin (109)	
Arctica islandica (B)		Ampharete baltica (P)	Bylgides sarsi (P)	
Bylgides sarsi (P)		Astarte borealis (B)	Diastylis rathkei (C)	
Diastylis rathkei (C)		Diastylis rathkei (C)	Macoma balthica (B)	
Heteromastus filiformis (P)		Macoma balthica (B)	Scoloplos armiger (P)	
Lagis koreni (P)		Mya arenaria (B)		
Nephtys hombergii (P)		Mytilus edulis (B)		
		Peringia ulvae (G)		
		Pygospio elegans (P)		
		Scoloplos armiger (P)		

The statistical significance and importance of environmental variables in explaining the temporal variability of macrozoobenthic data differed between the stations and depending on the biotic parameter considered. According to DistLM results, overall benthic biology in Mecklenburg Bight (012) was mainly driven by oxygen deficiency and the salinity regime in the year before the actual sampling (Table 4). The NAO index showed a significant contribution only for Pilou and Shannon anomaly (Table 4). A substantial decline in community parameters at station 012 corresponds to the oxygen minimum values (e.g. in years 1983, 1986 and 2002, Figs 7 and 8). On the other hand, the benthic indices are doubled or tripled (e.g. 1997 and 2009) in some years with usual oxygen conditions. However, the behaviour of the community parameters and their derivates is not always consistent. For instance in 2007, although abundance, biomass and species number were increased and no oxygen deficiency was indicated, the diversity indices reacted inconsistently. In the year 2000 with relatively low oxygen minimum (1.26 ml/l), abundance, biomass and species number were elevated, whereas the diversity indices were mainly distinctly below the median value.

**Fig 7:**
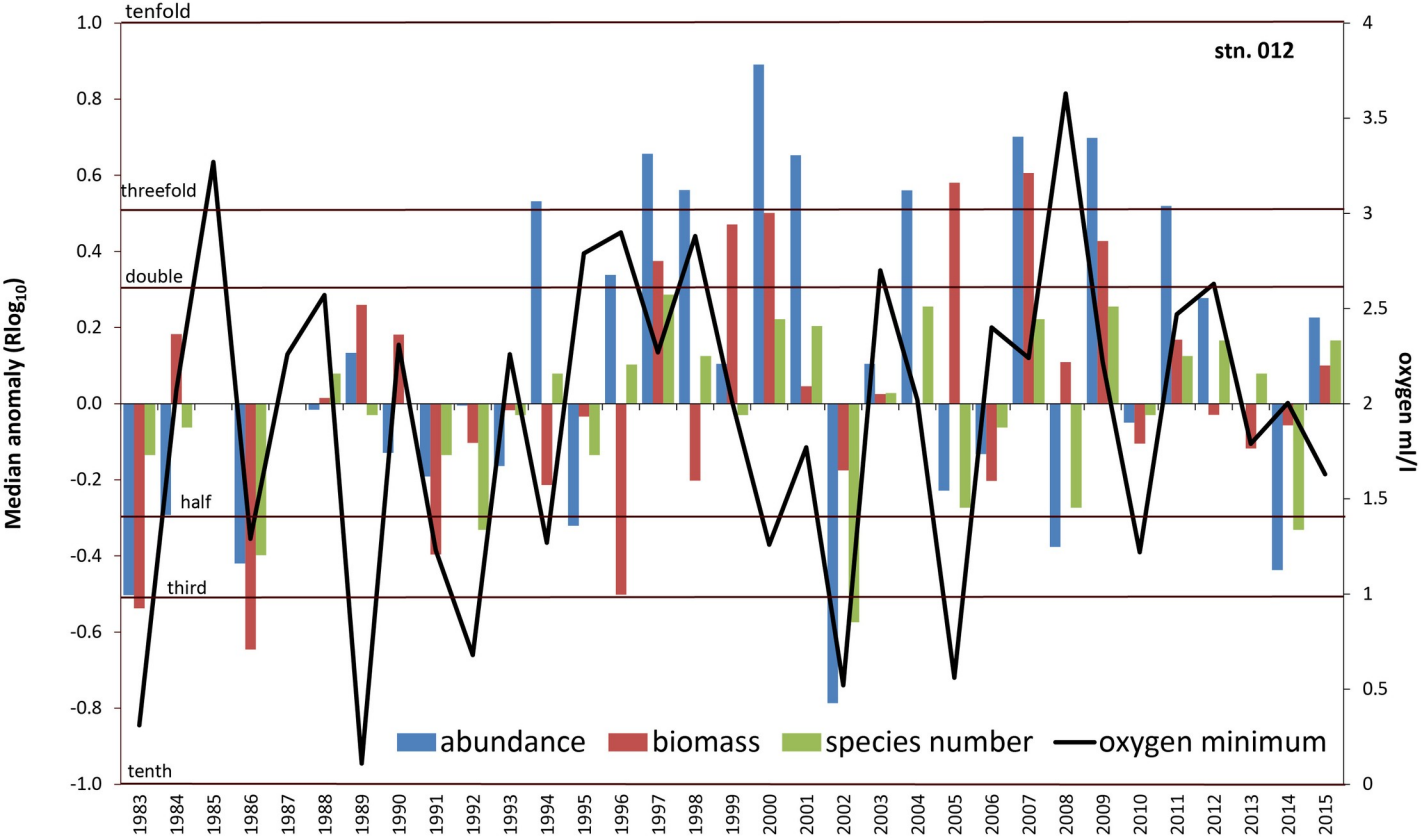
Median anomaly of abundance, biomass and species number at station 012. Logarithm to base 10 of ratio between annual and median value of the long-term data (left axis). Negative anomaly means annual value is smaller than median value and vice versa. Multiples of the median are indicated by dotted lines. Solid line = Oxygen minimum value per year (right axis).

**Fig 8:**
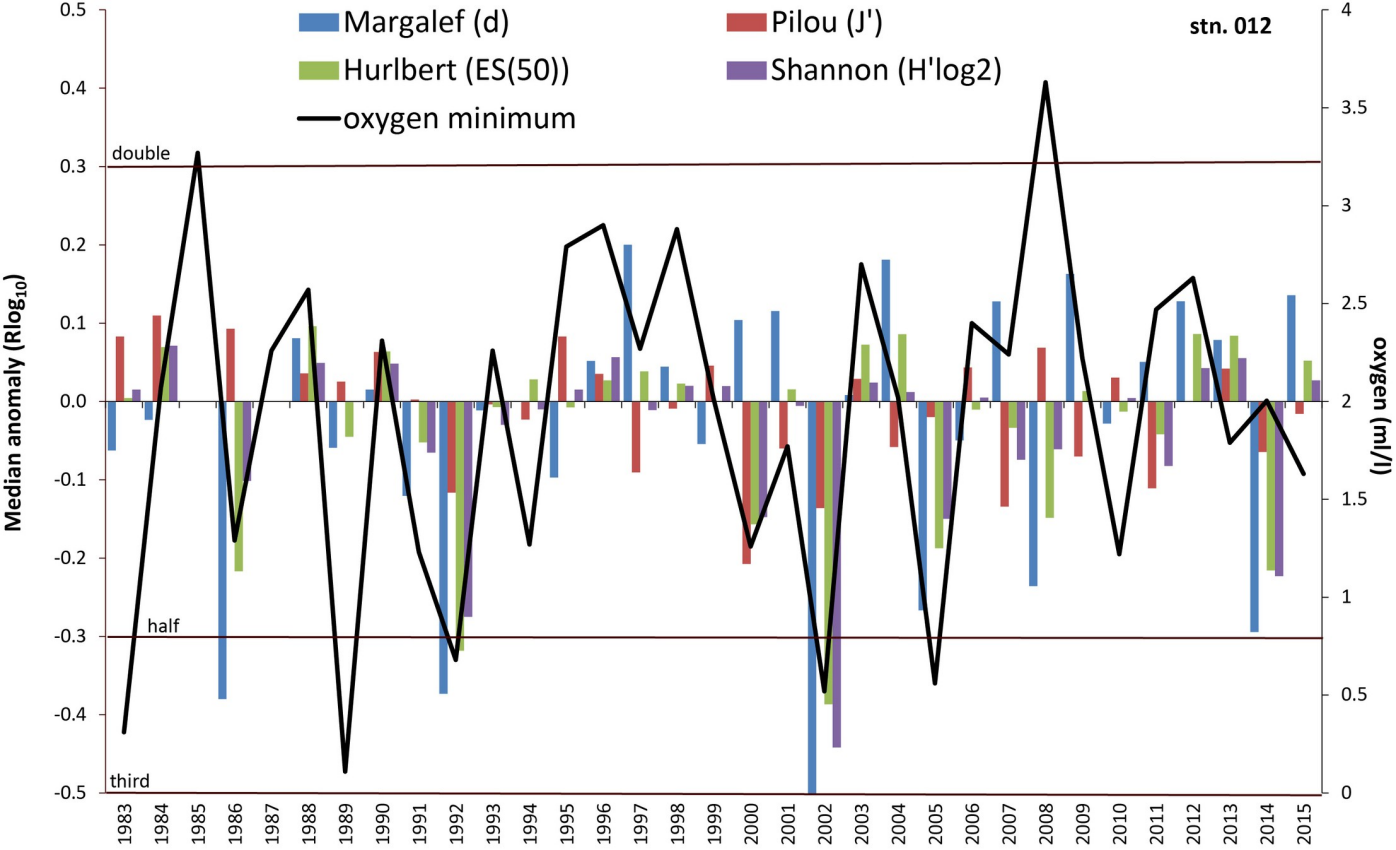
Median anomaly of diversity indices at station 012. Logarithm to base 10 of ratio between annual and median value of the long-term data (left axis). Negative anomaly means annual value is smaller than median value and vice versa. Multiples of the median are indicated by dotted lines. Solid line = Oxygen minimum value per year (right axis).

**Table 4:** Distance-based linear model (DistLM) of macrozoobenthos parameters and diversity indices against abiotic factors for station 012 (Mecklenburg Bight). Predictor variables subjected to a sequential stepwise selection procedure using the AICc criterions.

Response	Variable	AICc	SS(trace)	Pseudo-F	P	Prop.	Cumul.	res.df
*abundance*	*oxygen-days below 2 ml/l*	-5.4	5.69	7.37	0.013	0.21	0.21	28
* *	*oxygen-minimum previous year*	-7.9	3.32	4.90	0.036	0.12	0.33	27
*biomass*	days with salinity below 10th-percentile previous year*	-28.4	1.94	5.43	0.034	0.16	0.16	28
* *	oxygen-days below 50%*	-30.7	1.46	4.60	0.043	0.12	0.28	27
*species number*	*no significant model found*							
*abundance-anomaly*	*oxygen-days below 2 ml/l*	-55.3	1.08	7.37	0.010	0.21	0.21	28
	*oxygen-minimum previous year*	-57.8	0.63	4.89	0.037	0.12	0.33	27
*biomass-anomaly*	oxygen-days below 50%*	-73.4	0.42	5.21	0.031	0.16	0.16	28
	days with salinity below 10th-percentile previous year*	-75.7	0.33	4.70	0.039	0.12	0.28	27
*species number-anomaly*	*oxygen-days below 2 ml/l*	-92.8	0.20	4.68	0.039	0.14	0.14	28
	*oxygen-minimum*	-95.5	0.19	5.11	0.036	0.14	0.28	27
*Margalef-anomaly *	*oxygen-minimum*	-104.4	0.16	5.77	0.021	0.17	0.17	28
	*oxygen-days below 2 ml/l*	-106.7	0.12	4.68	0.038	0.12	0.29	27
*Pilou-anomaly*	*NAOI (Winter) previous year*	-156.2	0.03	6.03	0.021	0.18	0.18	28
	oxygen-days below 50%*	-161.4	0.03	7.88	0.010	0.19	0.36	27
*Hurlbert-anomaly*	*oxygen-minimum*	-131.1	0.11	9.67	0.003	0.26	0.26	28
*Shannon-anomaly*	*oxygen-minimum*	-136.9	0.10	10.01	0.004	0.26	0.26	28
	*NAOI (Winter) previous year*	-139.2	0.04	4.64	0.040	0.11	0.37	27
	oxygen-days below 50% previous year*	-141.0	0.03	4.26	0.050	0.09	0.46	26

^*—variable was square root transformed before the analysis^

**Fig 9:**
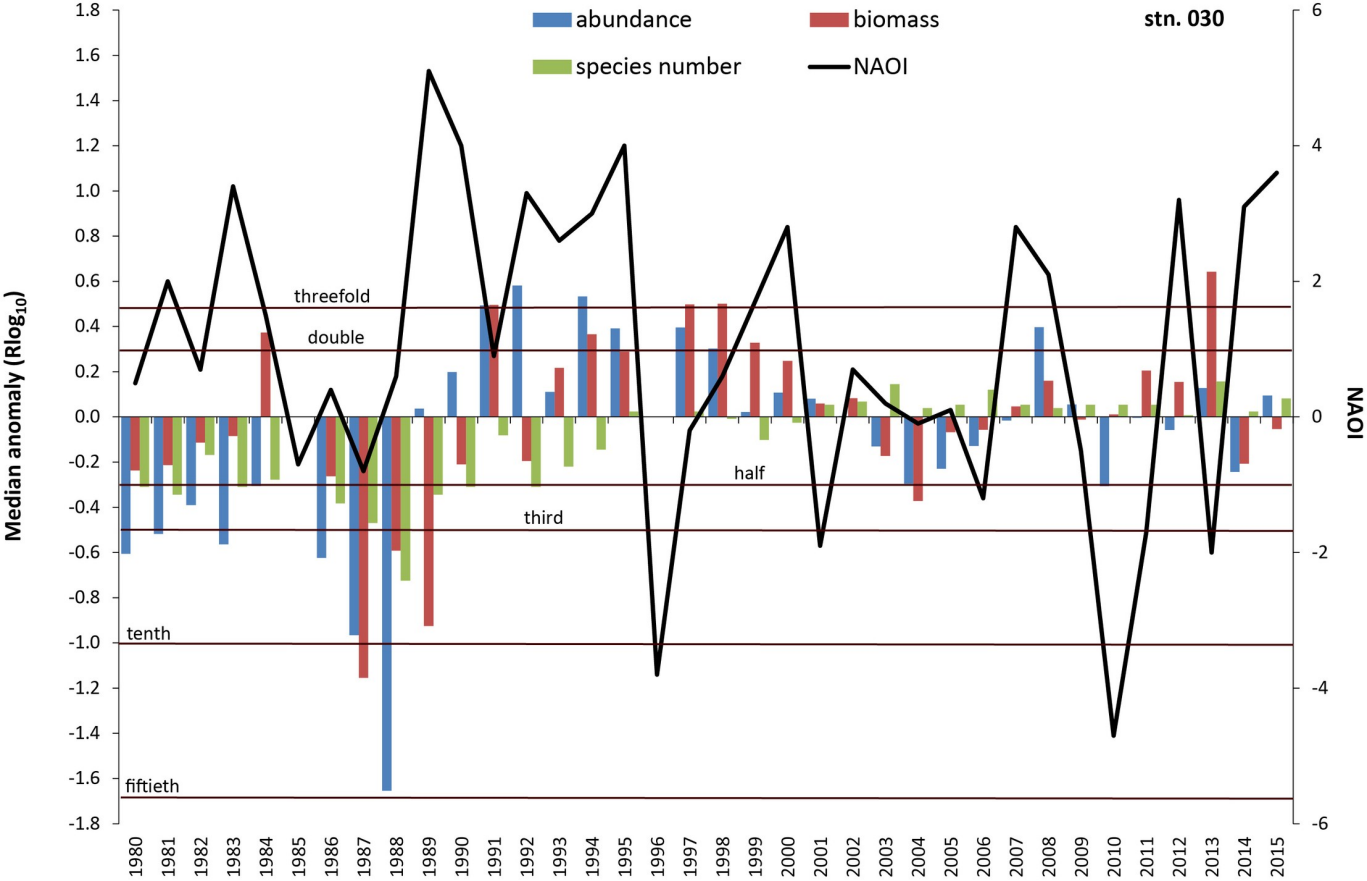
Median anomaly of abundance, biomass and species number at station 030. Logarithm to base 10 of ratio between annual and median value of the long-term data (left axis). Negative anomaly means annual value is smaller than median value and vice versa. Solid line = North Atlantic Oscillation Index (NAOI) for winter (DJFM) (right axis).

**Fig 10:**
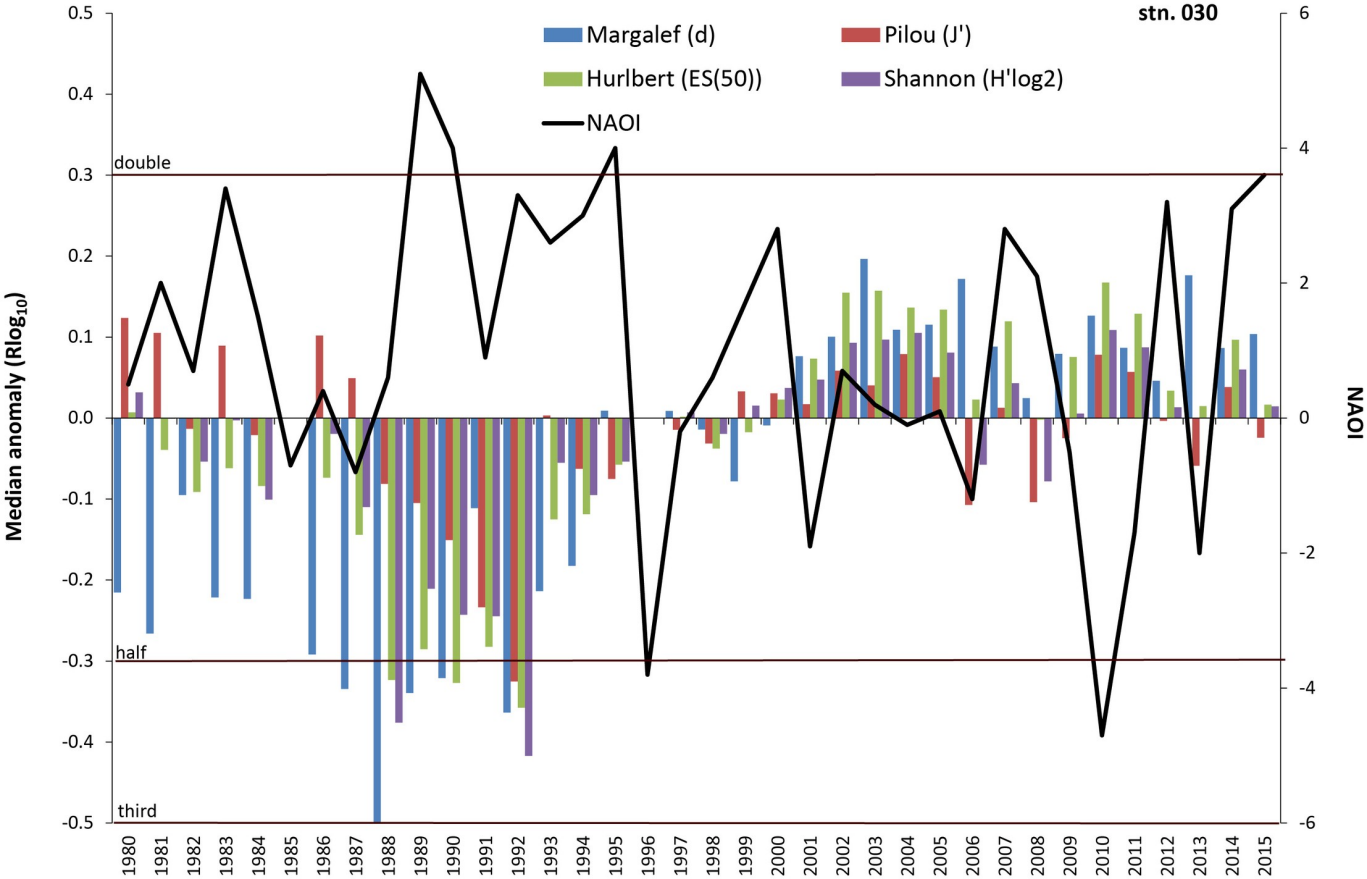
Median anomaly of diversity indices at station 030. Logarithm to base 10 of ratio between annual and median value of the long-term data (left axis). Negative anomaly means annual value is smaller than median value and vice versa. Solid line = North Atlantic Oscillation Index (NAOI) for winter (DJFM) (right axis).

At the Darss Sill (station 030) a significant part of the variability of all basic macrozoobenthic parameters could be explained by salinity conditions, especially in periods with extremely high or low values (Table 5). Increased salinity, reduced species abundance and biomass were characteristic for the first and the third quarters of the monitored period, whereas the second and the last quarters showed more days with salinity below the 10th-percentile and increased species density (Fig 5). The oxygen depletion in the previous year contributed most to the variability in species number and some of its derived indices. NAO index showed a significant negative correlation with species number and the derived indices (Figs 9 and 10). For Pilou’s evenness index, the NAO index of the previous year was most important (Table 5). Considering both—the previous and the actual year, a relation of NAO index with other basic variables also seems to exist. Yet, the time lags such as in the mid-1980s interfere with the overall statistical correlation.

**Table 5:** Distance-based linear model (DistLM) of macrozoobenthos parameters and diversity indices against abiotic factors for station 030 (Darss Sill). Predictor variables subjected to a sequential stepwise selection procedure using the AICc criterions.

Response	Variable	AICc	SS(trace)	Pseudo-F	P	Prop.	Cumul.	res.df
*abundance*	days with salinity below 10th-percentile previous year*	-1.4	6.96	7.79	0.007	0.20	0.20	31
*biomass*	days with salinity below 10th-percentile*	-23.7	5.58	12.27	0.002	0.28	0.28	31
* *	*Salt monthly anomaly annual maximum previous year*	-26.4	2.04	5.07	0.029	0.10	0.39	30
*species number*	oxygen-days below 50% previous year*	130.5	1052.40	21.67	0.000	0.41	0.41	31
* *	days with salinity below 10th-percentile*	126.7	258.28	6.21	0.020	0.10	0.51	30
	days with salinity above 90th-percentile previous year*	124.8	158.49	4.22	0.047	0.06	0.57	29
	days with salinity below 10th-percentile previous year*	122.1	167.43	5.09	0.031	0.07	0.64	28
*abundance-anomaly*	days with salinity below 10th-percentile previous year*	-56.3	1.32	7.79	0.009	0.20	0.20	31
*biomass-anomaly*	days with salinity below 10th-percentile*	-69.9	1.33	11.89	0.002	0.28	0.28	31
* *	*days with salinity above 90th-percentile*	-72.2	0.47	4.64	0.037	0.10	0.37	30
*species number-anomaly*	oxygen-days below 50% previous year*	-113.3	0.46	15.44	0.000	0.33	0.33	31
* *	days with salinity below 10th-percentile*	-118.3	0.19	7.52	0.010	0.13	0.47	30
	*days with salinity above 90th-percentile previous year*	-122.7	0.14	6.89	0.014	0.10	0.57	29
	days with salinity below 10th-percentile previous year*	-126.1	0.10	5.75	0.026	0.07	0.64	28
*Margalef-anomaly*	oxygen-days below 50% previous year*	-124.0	0.48	22.14	0.000	0.42	0.42	31
* *	days with salinity below 10th-percentile*	-126.9	0.10	5.30	0.029	0.09	0.50	30
* *	*days with salinity above 90th-percentile previous year*	-135.8	0.17	12.02	0.002	0.15	0.65	29
* *	days with salinity below 10th-percentile previous year*	-138.2	0.06	4.75	0.037	0.05	0.70	28
	*NAOI (Winter)*	-140.8	0.05	5.00	0.034	0.05	0.75	27
*Pilou-anomaly*	*NAOI (Winter) previous year*	-158.5	0.05	6.62	0.016	0.18	0.18	31
	*Salt monthly anomaly annual maximum*	-163.0	0.04	7.00	0.015	0.16	0.33	30
	*Salt monthly anomaly annual mean previous year*	-166.6	0.03	5.98	0.018	0.11	0.45	29
*Hurlbert-anomaly*	oxygen-days below 50% previous year*	-136.5	0.25	16.96	0.001	0.35	0.35	31
	*days with salinity above 90th-percentile previous year*	-141.6	0.09	7.65	0.010	0.13	0.48	30
	days with salinity below 10th-percentile*	-148.0	0.09	9.12	0.005	0.12	0.61	29
	*NAOI (Winter)*	-152.0	0.05	6.39	0.018	0.07	0.68	28
*Shannon-anomaly*	oxygen-days below 50% previous year*	-141.1	0.14	10.70	0.004	0.26	0.26	31
	*Salt monthly anomaly annual mean previous year*	-144.2	0.06	5.55	0.019	0.12	0.37	30
	*NAOI (Winter) previous year*	-146.6	0.05	4.71	0.036	0.09	0.46	29
	*Salt monthly anomaly annual maximum*	-147.8	0.03	3.62	0.048	0.06	0.52	28

^*—variable was square root transformed before the analysis^

In the Arkona Basin (station 109) the smallest number of significant correlations between the response parameters and the explanatory variables was detected (Table 6). For variations of abundance, biomass, Shannon-diversity and Pilou’s evenness no significant DistLM model could be derived. In contrast, variability in species number and the remaining indices revealed significant correlations with oxygen related variables, NAO index and salinity. In general, oxygen condition in the year of the sampling event was the most important parameter. Yet, despite the expectations, an observed significant positive temporal trend of the number of days with reduced oxygen (Pearson R = 0.70, p<0.001) coincided with significant, though less pronounced, surprising increase of species number (Pearson R = 0.62, p<0.001).

**Table 6:** Distance-based linear model (DistLM) of macrozoobenthos parameters and diversity indices against abiotic factors for station 109 (Arkona Basin). Predictor variables subjected to a sequential stepwise selection procedure using the AICc criterions.

Response	Variable	AICc	SS(trace)	Pseudo-F	P	Prop.	Cumul.	res.df
*abundance*	*no significant model found*							
*biomass*	*no significant model found*							
*species number*	*oxygen-days below 50%*	80.4	86.74	6.99	0.013	0.19	0.19	29
* *	days with salinity below 10th-percentile*	77.0	61.72	5.80	0.022	0.14	0.33	28
* *	*NAOI (Winter) previous year*	70.6	75.48	9.16	0.005	0.17	0.50	27
*abundance-anomaly*	no significant model found							
*biomass-anomaly*	no significant model found							
*species number-anomaly*	*oxygen-days below 50%*	-106.9	0.20	6.84	0.015	0.19	0.19	29
	*NAOI (Winter) previous year*	-110.5	0.15	6.07	0.019	0.14	0.33	28
* *	days with salinity below 10th-percentile*	-115.6	0.16	7.70	0.011	0.15	0.48	27
*Margalef-anomaly*	*oxygen-days below 50%*	-110.6	0.14	5.45	0.027	0.16	0.16	29
	*Salt monthly anomaly annual maximum*	-113.9	0.13	5.71	0.023	0.14	0.30	28
* *	*NAOI (Winter) previous year*	-116.6	0.10	5.16	0.032	0.11	0.41	27
*Pilou-anomaly*	*no significant model found*							
*Hurlbert-anomaly*	*oxygen-days below 50%*	-118.6	0.14	6.71	0.014	0.19	0.19	29
* *	*NAOI (Winter) previous year*	-121.3	0.09	5.05	0.035	0.12	0.31	28
*Shannon-anomaly*	*no significant model found*							

^*—variable was square root transformed before the analysis^

**Fig 11:**
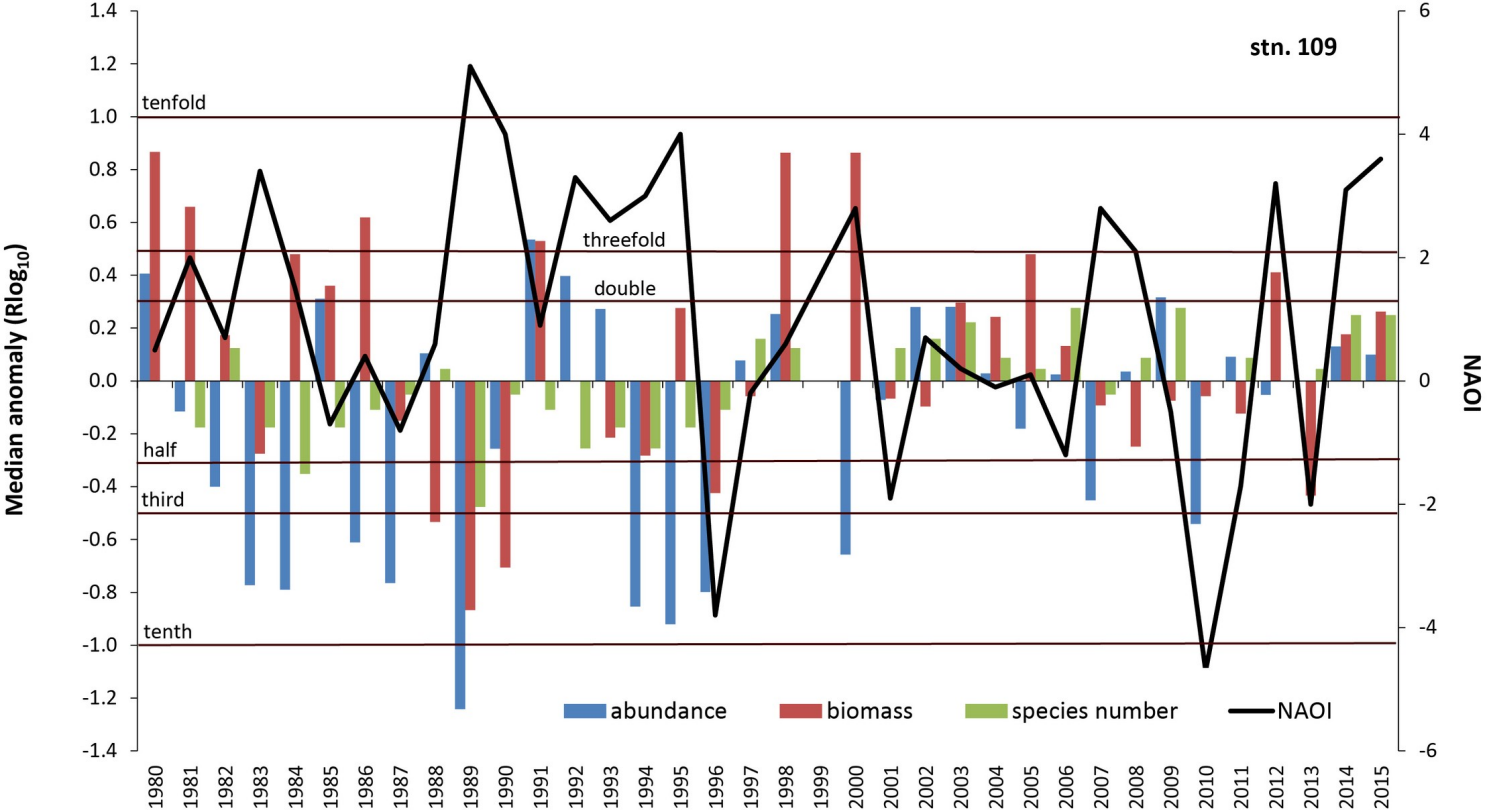
Median anomaly of abundance, biomass and species number at station 109. Logarithm to base 10 of ratio between annual and median value of the long-term data (left axis). Negative anomaly means annual value is smaller than median value and vice versa. Solid line = North Atlantic Oscillation Index (NAOI) for winter (DJFM) (right axis).

**Fig 12:**
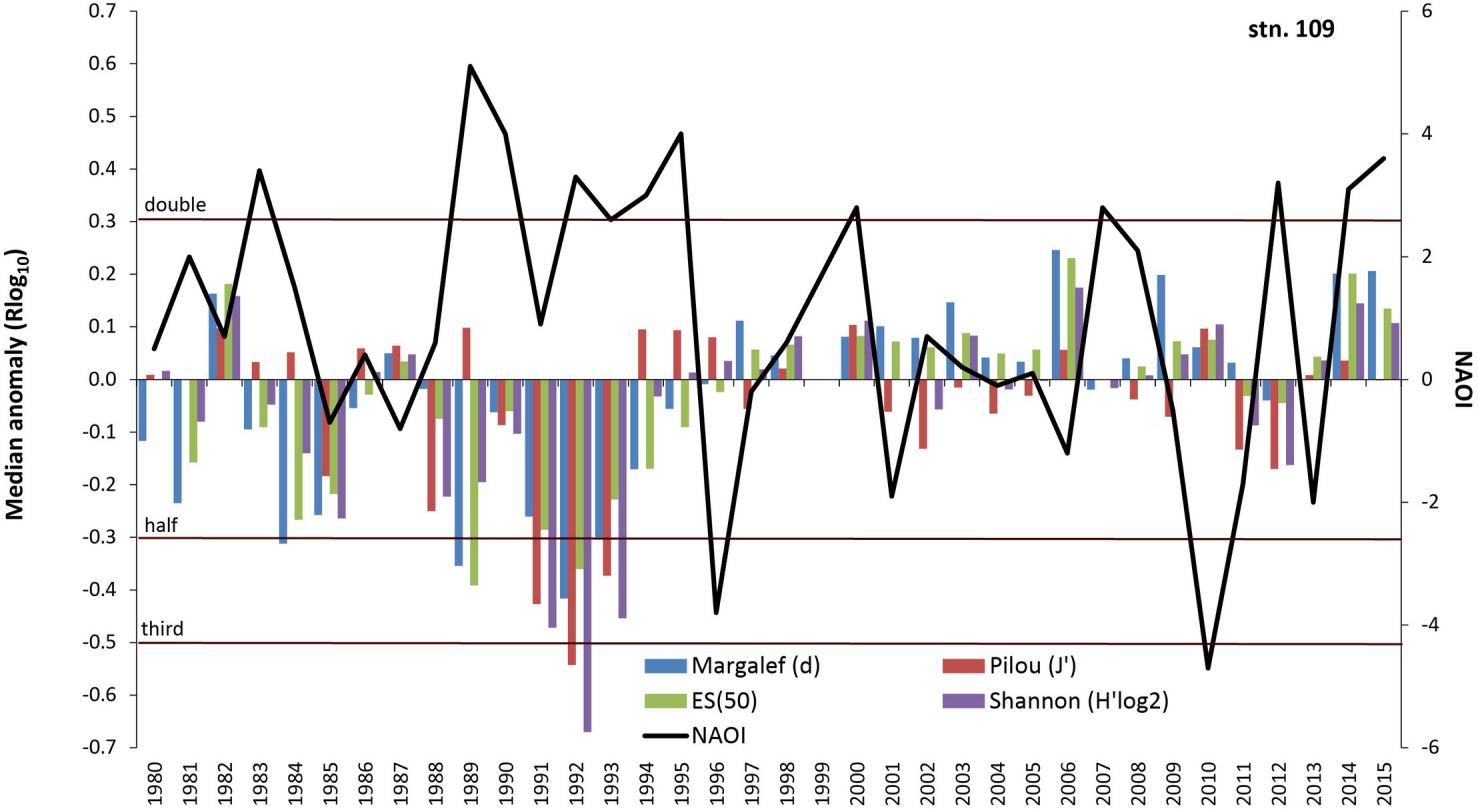
Median anomaly of diversity indices at station 109. Logarithm to base 10 of ratio between annual and median value of the long-term data (left axis). Negative anomaly means annual value is smaller than median value and vice versa. Solid line = North Atlantic Oscillation Index (NAOI) for winter (DJFM) (right axis).

The correlation of macrobenthic parameters and the NAO index is not consistent over the whole time span (Figs 11 and 12). Especially in the 1980s and 1990s, the behaviour of species number, Margalef and Hurlbert indices is strongly negatively related to the NAO index, whereas in the second half of the investigation period this relationship weakens, and the only significant positive correlation was found for biomass (R = 0.44, p<0.1).

## Discussion

Although at first glance the variation in basic macrozoobenthic parameters appears to be chaotic, we could reveal significant influences of different environmental and climatic variables on the temporal variability of macrozoobenthos using statistical approaches. However, the main lesson learned from this study is that even at a restricted spatial scale, the benthic system does not appear to be tightly controlled by any single driver. In contrast, the influence of the individual environmental factors (and consequently also of anthropogenic pressures) on the species assemblages of different habitats is rather dissimilar. Additionally, the strength of the linkages with investigated environment drivers varies between the considered macrobenthic parameters.

### Relevance of selected environmental parameters

**Fig 13:**
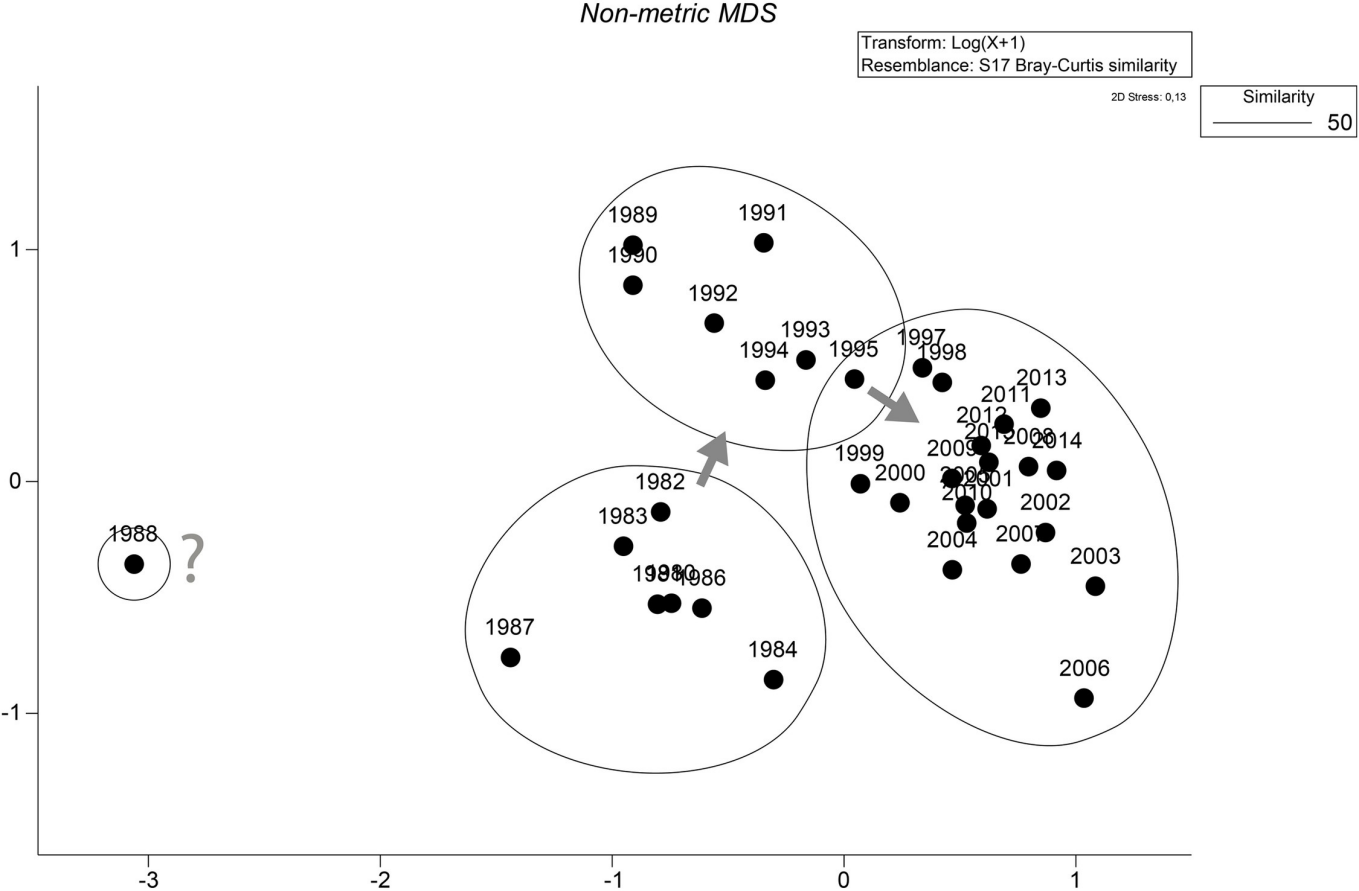
Ordination of Bray-Curtis similarities in species composition and abundance of the station 030 from 1980 to 2015. Arrows indicate strong changes and might be seen as “regime shifts”.

Climate and related factors such as in- and outflow situations, stagnation periods and change of eutrophication level via temperature, precipitation and river runoff, salinity and oxygen conditions significantly influence the benthic life in many coastal waters around the world (e.g., [14, 15, 21, 45–47]) and many of these factors are interrelated. Moreover, the enrichment of nutrients is evident along the world’s coastlines (see [48]), including effects like the sixfold expansion of the hypoxic zones in the Baltic Sea from 1950 to 2000 [12, 49]. A cross-realm assessment of climate change impacts on species’ abundance trends shows a consistent effect of temperature change in the terrestrial communities and more variable effects in the aquatic communities [50]. Model simulations for the south-western Baltic Sea showed a doubling of summer Chlorophyll-a and oxygen stress compared to the pre-industrial situation [32]. Recent analysis of the North Sea data revealed that the linear relationship between the NAO winter index and benthic macrofauna failed to achieve statistical significance after 2000 with the conclusion that the NAO index is no longer seen as suitable climate predictor [51, 52]. Nevertheless, we could show that at least in some areas of the western Baltic Sea the NAO winter index explains up to 18% of the median anomaly of diversity indices. However, our results also suggest that the relationships between the NAO and diversity indices seem to be much less pronounced after the second millennium. Until end of the 1990s, an oscillatory pattern with cycles of seven years is observed at Darss Sill (Figs 9 and 10), which is very similar to the pattern reported for benthic macrofauna from the northern Baltic Sea [53] and from the Skagerrak area [46]. Yet it remains inexplicable why these periods are no longer visible on the ordination diagram after the 1990s (Fig 13). Thus significant correlations between benthic community parameters and the NAO index are found (Tables 4 to 6), but the strength and also the temporal lag of these correlations are variable and difficult to interpret [46].

Salinity is considered the key factor for community changes at different spatial scales along the Baltic Sea [6, 10]. Our results confirm that on temporal scales too variability in salinity is an important driver for variation in the benthic community. As expected, its influence, especially for abundance and biomass changes, is highest at the Darss Sill with highest variability in salinity. Rapid changes in salinity represent severe deviation in environmental conditions and can provoke physiological stress especially for the dominating brackish water species leading to mortality, reduced fertility or active avoidance behaviour [8].

Often oxygen depletion, considered among the most widespread deleterious influences on marine benthic environments [54], overrides all other signals forced by different drivers. In our study oxygen variables performed best to explain the fluctuations of most macrobenthic parameters in the Mecklenburg Bight and Arkona Basin. Rather unexpectedly, the number of days with oxygen below 50% for the previous year also explained over 40% of variation in species number at Darss Sill.

The hydrodynamic regime is characterised by a number of physical properties and circulation patterns, whose essential variability itself is partly affected by climate change. However, these effects can only rarely be separated from the natural variation [14]. Long-term field studies of benthos in the Baltic Sea have tended to identify a critical role of winter conditions, particularly temperature, in macrozoobenthic dynamics of offshore stations, whereas the signal in near coastal waters was overwhelmed by anthropogenic influence of eutrophication indicated by nutrient inputs [45]. In highly complex transitional ecosystems, such signals of anthropogenic disturbance can be confounded by the variability of climate through its separate influence over watersheds and ocean basins [48]. However, clear signals can emerge when time series are extended long enough and include measurements (or model estimates) of key drivers of change in benthic communities (e.g., [11]). Studies from the North Sea showed strong effects of winter physical conditions on the interannual and long-term variation of macrofaunal biomass [19, 55]; changes in species composition were driven by fishing impacts, climate warming and altered downward fluxes of phytoplankton [22].

Multivariate analysis was employed to visualise trends in community composition. Whereas for the muddy stations in the Mecklenburg Bight (012) and Arkona Basin (109), frequently influenced by oxygen deficiency, no “regime shifts” and no clear trends could be observed, the benthic community at the Darss Sill (030) changed significantly several times over the reflected time period (Fig 13). The community seems to undergo quasi-stable periods with interannual variations alternated by more abrupt shifts. Two major shifts (around the end of 1980s and around mid-1990s) were observed and might be seen as benthic, lagged, responses to the now widely accepted North Atlantic “regime shift”. Such shifts have already been found for several components of the marine ecosystem: fish (e.g., [56, 57]), phytoplankton (e.g., [58]), zooplankton (e.g., [59]) and also benthos (e.g., [30]). This marked change is further apparent in plankton records from a wide area of the north-eastern Atlantic and appears to be linked to changes in atmospheric dynamics/meteorology, as indicated by the NAO index [22].

### Uncertainties in measurements and modelling

Any measured or model-based calculated parameter incorporates uncertainties. Measurements are unable to provide full spatial and temporal coverage, while models are simplifications of the complex environment. In response to driving forces, the benthic communities comprise a broad view by integrating not only the present situation, but also previous conditions. According to our results, in 2012 the minimum oxygen concentration at station 012 was measured with 2.65 ml/l, while the model simulation predicted more than 150 days below 1 ml/l. Although there was no measurement between August (2.65 ml/l) and November (3.51 ml/l), the unusually long period with low oxygen in the model seems to be an overestimation of the reality. Data from the more nearshore station 022 (Lübeck Bay, monitored regularly by German environmental authorities) for 2012 shows the same annual cycle as in the other years, with values near to zero in August and September. This reveals that in Mecklenburg Bight low oxygen conditions may have also occurred. On the other hand, the benthic community from autumn 2012 showed no relevant disturbance, indicating that no severe oxygen stress occurred. Such discrepancy emphasizes the need for caution when working with model results, but also suggests that incorporating data of the benthic communities can substantially enhance the quality of ecosystem models.

All these aspects add to the complexity of the system and the result´s interpretation. The outcome of our analysis reflects some general principles of how abiotic variables drive the high temporal fluctuations of diversity, abundance and biomass of macrozoobenthos. Thereby, the characteristics of the habitat itself are a separate factor. The unexplained variation in turn refers to the limitations of the available data as well as to the coincidence of stochastic processes. Other principles that drive the distribution of macrofauna manifest themselves best when the variations on the large spatial scales are in focus (e.g. [60]). Such principles or strategic relationships remain central in explaining the large scale phenomena of species’ succession or evolution. Alternatively, the chaos theory may help to deal with a complex system whose behavior is highly sensitive to slight changes, or the Bayesian hierarchical spatial-temporal approach can be used when spatially varying temporal trends along with spatially correlated random processes are assumed [61].

### Relevance for assessment of anthropogenic impacts

Our results clearly indicate that even within a narrow spatial scale, benthic communities are influenced by different natural regimes leading to dissimilar exposure to natural stress. These different natural boundary conditions may lead to various genetic or phenotypic adaptations of key species between regions [62] which in turn may be followed by distinct behaviours of benthic communities during additional anthropogenic disturbance. Thus, the transfer of the assessment of e.g. the sensitivity of benthic communities against specific anthropogenic impacts should be done with caution and with consideration of the natural boundary, especially in regions with high natural environmental variability, such as transitional areas. Further, this study highlights the importance of keeping long-term data series alive, even if the requirements of many monitoring programs such as those implemented under the framework of the European MSFD, focus on a spatial approach with lower intensity per station.

## Conclusion

Given the important ecological role of benthic communities, it is essential to understand and predict their response to climate change and other anthropogenic or natural pressures. Some taxa are more vulnerable than the other, so different species-specific effects may lead to major changes in the composition of the benthic community. Long-term data sets are indispensable in assessing the state of benthic systems and ecological processes within it, to disentangle human-induced and natural changes, short-term fluctuations and long-term trends [11, 51, 63]. This highlights the importance of maintaining long-term monitoring programs. Thinking from a management perspective, it is crucial to know if changes in the environment with all subsequent alterations are man-made or ranging within their natural spectrum. Thereby it is a crux that nowadays undisturbed areas almost do not exist and human pressures interfere with the effects of both natural variability and climate changes [64]. Many anthropogenic pressures, such as eutrophication, ocean acidification or bottom trawling, are not considered in the present study. Nevertheless, our results provide some insight into the ecological dynamics of a coastal ecosystem naturally exposed to a high degree of spatio-temporal variability. Here, we have considered only few abiotic parameters and one climate proxy. Employing a statistical approach, the long-term development of basic macrozoobenthic parameters (species richness, abundance and biomass) and its derivates could be interpreted and partly explained within the context of the interrelationship between environmental and climatic changes. To conclude, the interpretation of variation of benthic communities reflects the complexity contained by the data, and therefore is it always a challenge, but not a speculation.

## Supporting information

S1 File Derivative indices used. (DOCX)

S1 Table List of abiotic variables. (XLSX)

S2 Table Supplementary data. (XLSX)
