# Supplementary material for: Variation in benthic long-term data of transitional waters: Is interpretation more than speculation?
Source: PLoS One. 2017 Apr 19;12(4):e0175746. doi: 10.1371/journal.pone.0175746 (PMC5396916; doi:10.1371/journal.pone.0175746)
Supplement: S1 File — (DOCX) [file pone.0175746.s001.docx]

**Supporting information 1. Derivative indices used**

The most commonly used diversity index is Shannon (or Shannon-Wiener) species diversity index (H'log_2_) [36, 37]:

$H^{'}=-\sum_{i} p_{i}\log_{2}(p_{i})$,

where $p_{i}$ is the proportion of the total count (abundance) arising from the *i*th species. The logarithm to base 2 used here corresponds to the measurement units that reflect the amount of information that can be attributed to each individual.

Margalef ‘s index ($d$) incorporates the total number of species (S) and the total number of individuals ($N$) and is a measure of the number of species present for a given number of individuals [38]:

$$d=(S-1)/\ln N$$

Equitability is expressed as Pilou’s evenness index (J') [39]:

$J^{'}={H^{'}}/{{H^{'}}_{max}}={H^{'}}/{\log_{2} S}$,

where ${H^{'}}_{max}$ is the maximum possible value of Shannon diversity that would be achieved if all species were equally abundant.

The Hurlbert index is designed to generate an absolute measure of species richness comparable across samples of different size [40]. It gives the estimated species number of a randomly picked subset of 50 individuals (ES_(50)_) at the sampling site:

$${ES}_{(50)}= \sum_{i=1}^{S} \left[ 1-\frac{\left( N-N_{i} \right)!\left( N-50 \right)!}{\left( N-N_{i}-50 \right)!N!} \right]$$
